# Field Test of a Remote Multi-Path CLaDS Methane Sensor

**DOI:** 10.3390/s150921315

**Published:** 2015-08-28

**Authors:** Genevieve Plant, Michal Nikodem, Phil Mulhall, Ruth K. Varner, David Sonnenfroh, Gerard Wysocki

**Affiliations:** 1 Electrical Engineering Department, Princeton University, Princeton, NJ 08544, USA; E-Mails: gplant@princeton.edu (G.P.); mnikodem@princeton.edu (M.N.); 2 Wroclaw Research Centre EIT+, 54-066 Wrocław, Poland; 3 Physical Sciences Inc., 20 New England Business Center, Andover, MA 01810, USA; E-Mails: mulhall@psicorp.com (P.M.); sonnenfroh@psicorp.com (D.S.); 4 Institute for the Study of Earth, Oceans, and Space, and Department of Earth Sciences, University of New Hampshire, Durham, NH 03824, USA; E-Mail: ruth.varner@unh.edu

**Keywords:** optical dispersion spectroscopy, laser spectroscopy, remote sensing

## Abstract

Existing technologies for quantifying methane emissions are often limited to single point sensors, making large area environmental observations challenging. We demonstrate the operation of a remote, multi-path system using Chirped Laser Dispersion Spectroscopy (CLaDS) for quantification of atmospheric methane concentrations over extended areas, a technology that shows potential for monitoring emissions from wetlands.

## 1. Introduction

Methane is a potent greenhouse gas that is of importance in discussions about climate and energy. Both natural and anthropogenic sources of methane are of interest to the atmospheric science community. It is well known that natural methane sources are often spatially heterogeneous [1], which makes large area monitoring and the ability for spatial identification of increasing importance. Currently there are multiple technologies used to monitor atmospheric methane concentrations and the most accurate and widely accepted in the atmospheric research community are optical sensing technologies based on single location (extractive or open-path) laser absorption spectrometers [2,3]. While these techniques offer high sensitivities, the lack of spatial information limits their ability to identify sources or sinks without large infrastructures of multiple, often costly, sensor nodes. To address this need, a remote, multi-path sensor was designed, characterized [4], and deployed in the field. The results of this first field test were to illustrate the capability of this sensing configuration and indicate needs for further technology development. Use of a centralized system allows for distribution of the spectrometer cost over multiple sensing paths, effectively eliminating the need for a network of multiple sensor nodes. The location for this field campaign was Sallie’s Fen Environmental Station (SFES), a site monitored for methane emissions by the University of New Hampshire for more than two decades [5,6,7,8]. This long-term record of methane emissions and also meteorological variables measured at this wetland, located in Barrington, NH (43°12.5′ N, 71°3.5′ W), make this site well suited for studies of methane from wetland sources. Wetlands are the largest natural emitters of methane [8], and thus SFES is an important representative ecosystem and provides an excellent testing platform for evaluation of a new instrument.

## 2. Spectroscopic Sensing System

A technique known as Chirped Laser Dispersion Spectroscopy (CLaDS) was recently developed to address some of the key issues in traditional absorption-based, remote sensing techniques; namely the dependence of the measured signal amplitude on the received power and the linearity and dynamic range of concentration detection are significantly improved with CLaDS [9,10]. CLaDS takes advantage of measuring the optical dispersion induced by interaction of the laser light with the targeted species. This is achieved by probing the sample transition with a frequency-chirped, multi-color, coherent laser beam. Since each component wave (color) experiences a different index of refraction related to the dispersion profile of the molecular transition, the sample dispersion can be extracted through analysis of the instantaneous frequency of the heterodyne beat note generated by the component waves on a square-law photodetector. Unlike an absorption measurement, which is based on quantification of transmitted light intensity, a dispersion measurement is determined from the phase of the detected light and thus is fundamentally independent of the received optical power [11]. Key benefits of CLaDS, such as immunity of the CLaDS signal to intensity fluctuations, and a linear response with concentration [12], are achieved while still maintaining sensitivities needed for environmental trace gas monitoring [13]. All of these qualities are essential for open-path measurements where atmospheric conditions can vary drastically resulting in strong fluctuations of the intensity of the light received on the photodetector (e.g., due to fog or dust). To provide continuous monitoring functionality through spectral line-locking to the target transition, a variant of CLaDS technique, called Chirp Modulated (CM) CLaDS [11] was employed in this instrument.

In a recent work, the feasibility of CLaDS operation in the near-infrared was demonstrated, showing the use of both single sideband (SSB) and double sideband (DSB) modulation of the optical carrier used for generation of CLaDS signals [14]. The instrument core developed for this field campaign was detailed in a previous publication (see [4]) and utilized DSB CM-CLaDS to target methane in the near-IR at 6046 cm^−1^ (1653 nm).

**Figure 1:**
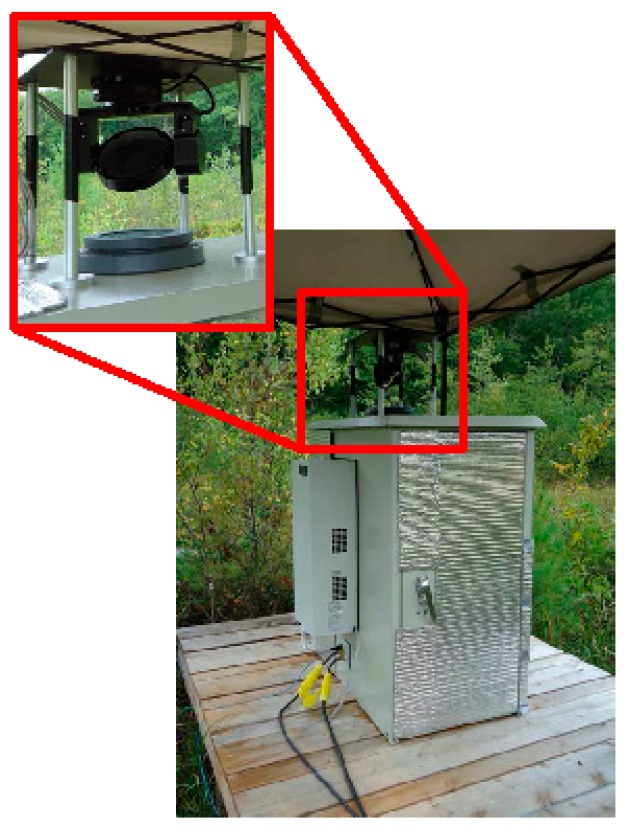
Chirped Laser Dispersion Spectroscopy (CLaDS) methane instrument installed in a weatherized enclosure; the inset shows a close-up of the optical port and a computer-controlled gimbal mount used to direct light horizontally to different sections of the fen.

**Figure 2:**
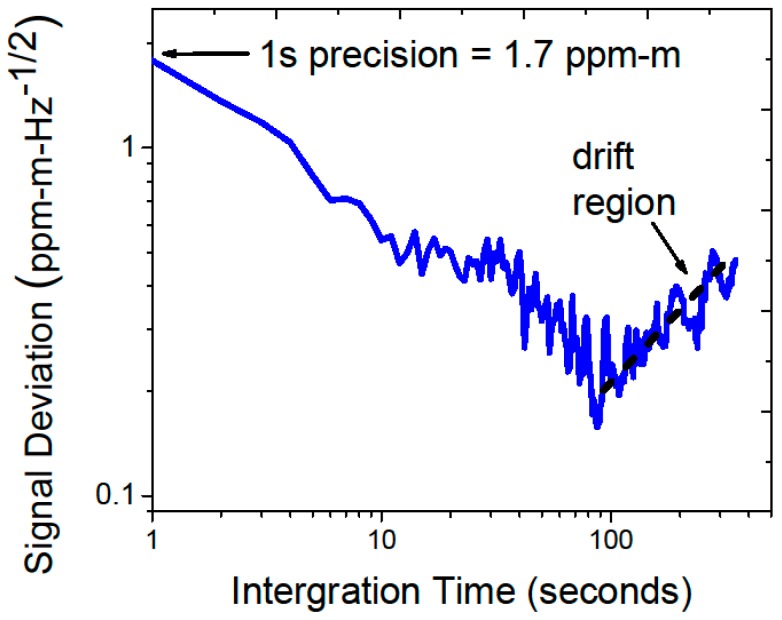
Allan Deviation analysis performed in the ambient methane conditions prior to transportation to Sallie’s Fen Environmental Station (SFES) shows a detection limit of 1.7 ppm-m-Hz^−1/2^ and system drift dominated performance for averaging times >100 s.

Prior to the field deployment, the system was installed in a weatherized, temperature-controlled cabinet (manufacturer: DDB Unlimited), shown in Figure 1. A sealed polycarbonate window was installed in the top of the cabinet to allow the laser light to be launched from inside the enclosure with negligible transmission loss (see inset in Figure 1). A gimbal mounted mirror system was used to direct the laser radiation to multiple retroreflectors located at a given distance away from the sensor cabinet. The system was characterized in ambient methane conditions prior to installation at SFES. With the assumption that the atmospheric methane concentration was constant during the test, a path-length and bandwidth normalized detection limit of 1.7 ppm-m-Hz^−1/2^ was achieved (Allan Deviation is shown in Figure 2), which was consistent with the specifications of the laboratory prototype tested earlier [4].

**Figure 3:**
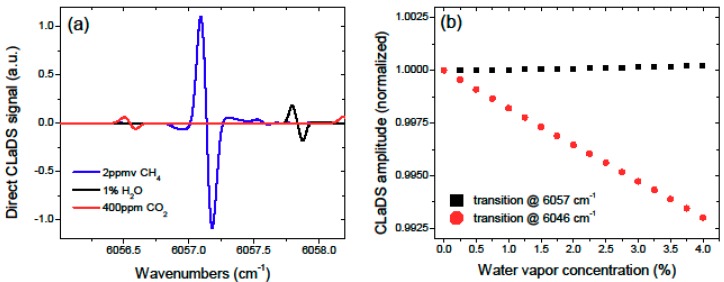
(**a**) Simulated CLaDS spectra of the three main atmospheric species in the 1651 nm (6057 cm^−1^) region; (**b**) Impact of the water vapor concentration on the peak amplitude of the CH_4_ CLaDS signal calculated for the transitions at 6057 and 6046 cm^−1^ (both have similar linewidths, thus double sideband (DSB) spacing of Ω = 1.2 GHz was used for calculating the CLaDS signal in both cases).

For the experiments shown in [4], the R3 transition in the 2ν_3_ band of methane at 6046 cm^−1^ (1653 nm) was used. Due to spectral interference from water vapor, we switched to the R4 transition in the same band at 6057 cm^−1^ (1651 nm), which provided better conditions for atmospheric open path measurements (the diode laser was operated at 5 °C instead of 25 °C used in [4]). This is illustrated in Figure 3, which shows the simulated CLaDS spectra for methane (CH_4_), water vapor (H_2_O), and carbon dioxide (CO_2_) around 6057 cm^−1^ based on the HITRAN 2008 database [15]. The cross-sensitivity of the CLaDS signal amplitude to atmospheric water concentration for the two CH_4_ lines is shown in Figure 3b, and clearly shows the influence of the close-by water transition on measurements performed at 6046 cm^−1^.

## 3. Deployment Details

**Figure 4:**
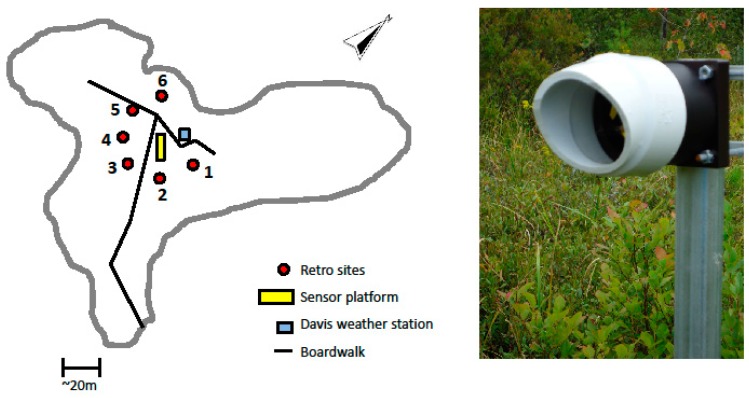
The sensor configuration deployed at SFES. The yellow rectangle, red circles, and the blue square represent the central sensor platform, the location of the six retro-reflectors, and the weather station, respectively. The image shows the mounting of the retro-reflector onto fence posts which were inserted several feet into the fen surface.

The CLaDS sensor was installed at SFES in late June 2013 with a sensing configuration as illustrated in Figure 4. Six 2-inch retro-reflectors were mounted in a star-like configuration ~20 m away from the sensor cabinet, and the locations were chosen to sample CH_4_ emissions from areas with different plant species. It should be noted that this distance of 20 m is not a maximum target distance, but was rather chosen to sample a certain area within the fen. The path length for each retro-reflector location was precisely measured and stored to be used in calculating the average methane concentration along each path. During the deployment each optical path was probed for two minutes, after which the gimbal position was changed to target the next reflector in the cycle.

**Figure 5:**
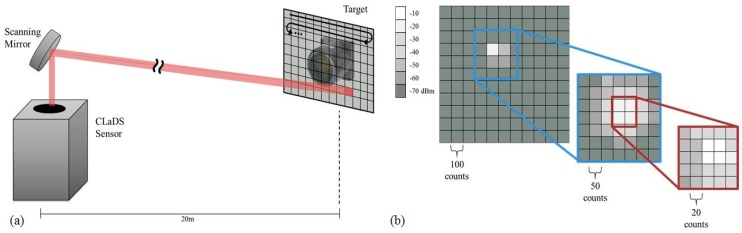
Raster algorithm used to optimize the scanning mirror position. (**a**) Configuration for the retro-reflector searching scheme; (**b**) Consecutive scans of decreasing grid size (11 × 11, 7 × 7, 5 × 5) and step size (100, 50, 20 counts) used maximize the return beatnote powers (in dBm) to the CLaDS sensor during initial position calibration.

The sensor platform and the retroreflector posts were affixed to the relatively unstable wetland surface, making it quite challenging to provide a stable optical alignment with the retroreflectors. To address this issue we developed an active alignment procedure with raster-scanning algorithms to realign the system before each two-minute sampling interval as well as aid in the initial alignment of the system (see Figure 5). Initial alignment of the system optics was performed using the scheme outlined in Figure 5b. A large grid and step size (11 × 11, 100 counts) was used for a rough alignment. The maximum of this scan was used as the center of another scan with smaller settings (7 × 7, 50 counts). Subsequently, the maximum of this scan was used as the center for fine tuning of the alignment (5 × 5, 20 counts). Once the maximum return signal position was identified the alignment algorithm set the mirror position at the same coordinates and the methane monitoring was initiated (the alignment algorithm was programmed to eliminate the effects of backlash from the gimbal motors). During the measurement cycle, if the return powers dropped below a set threshold, a raster scan was initiated at a small grid and step size settings (5 × 5, 20 counts). If after this first scan return powers did not meet the threshold condition, a second raster scan at larger settings (11 × 11, 100 counts) was performed. If again the threshold condition was not met, that path was flagged in the software as needing attention by a human operator.

A weather station (Davis Vantage Pro2, Barrington, NH, USA) was mounted several meters away from the cabinet and provided meteorological data (temperature, pressure, relative humidity, and wind speed and direction) collected with the methane signals. A 10 × 10 foot canopy was installed over the sensor platform to protect the setup from rain and to prevent direct sunlight from heating the cabinet. Optical powers of less than 1 mW were launched from the sensor with a divergent beam, making the sensing configuration eye-safe [16]. The system began collecting data in late July (30 July 2013) and continued until mid-October (21 October 2013), with a 3-week break in late August when the gimbal mount malfunctioned and was sent out for repairs.

### Signal Calibration in Field Conditions

**Figure 6:**
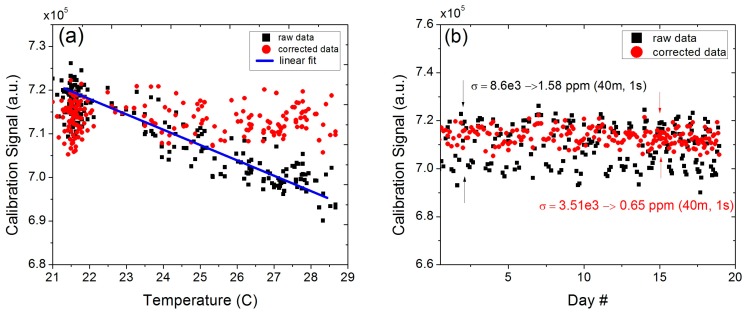
(**a**) Temperature dependence of the calibration signal as a result of larger temperature swings within the weatherized enclosure and the data corrected with by the temperature-dependent spectral model; (**b**) Calibration signals over the course of 19 days before and after temperature correction.

In order to ensure the system accuracy did not deteriorate during the deployment, a fiber-coupled gas cell (3 cm long and filled with 19.4% of methane in nitrogen at 740 Torr) was placed inside the cabinet to enable periodic re-calibration [4]. Despite being housed in an air-conditioned cabinet, the system experienced relatively larger temperature swings than planned (more than 7 °C) due to the limited functionality (lack of heating mode in the cool nights) and a moderate cooling capacity (4000 BTU) of the built-in air conditioning unit. As a result of the varying temperature in the cabinet, the raw calibration signal showed a clear temperature dependence (see Figure 6). Direct application of this raw calibration signal to the conversion of the acquired open-path data into concentration levels would introduce a large amount of error into the retrieved values.

To suppress the effects of cabinet temperature fluctuations, the raw calibration signal was corrected for both the temperature dependence of the molecular transition as well as for the temperature induced pressure changes within the sealed gas cell. The internal cabinet temperature was monitored continuously with the Davis Vantage Pro2 console temperature sensor and the data was used for correction of the calibration signal. The temperature correction of line intensity was determined using the following expression [17].

(1) $$S\left(T\right)=S\left(T_{ref}\right)\frac{Q\left(T_{ref}\right)}{Q\left(T\right)}\frac{\text{exp}\left(-\frac{c_{2}E_{\eta }}{T}\right)}{\text{exp}\left(-\frac{c_{2}E_{\eta }}{T_{ref}}\right)}\frac{\left[1-\exp\left(\frac{-c_{2}\nu_{\eta \eta^{\prime }}}{T}\right)\right]}{\left[1-\exp\left(\frac{-c_{2}\nu_{\eta \eta^{\prime }}}{T_{ref}}\right)\right]}$$

where *T* is the temperature (K), S(T) is the spectral line intensity [cm^−1^/(molecule cm^−2^)], Q(T) is the total internal partition function, *c*_2_ is the second radiation constant (1.4388 cm·K), $E_{\eta }$ is the lower state energy of the targeted transitions (cm^−1^), and $\nu_{\eta \eta^{\prime }}$ is the line frequency (cm^−1^). The above expression was used in conjunction with molecular parameters from the HITRAN documentation [18] to correct the change in line strength due to the deviation in temperature away from the reference temperature $T_{ref}$ used in HITRAN (usually a reference temperature and pressure of *T_ref_* = 296 K and *p_ref_* = 1 atm are used).

The effects of the change in pressure within the sealed cell were addressed using the ideal gas law in isovolumetric conditions yielding

(2) $$p=\frac{T}{T_{0}}p_{0}$$

where *p* and *T* are actual temperature and pressure in the cell (in (atm) and (K) respectively), and *p*_0_ and *T*_0_ are the nominal values provided by the manufacturer of the gas cell. The calculation of the actual pressure is performed based on the temperature measurements inside the sensor cabinet. Both temperature and pressure values were used to correct the pressure broadened halfwidth (cm^−1^/atm) according to [17],

(3) $$\gamma \left(p,T\right)={\left(\frac{T_{ref}}{T}\right)}^{n}(\gamma_{air}\left(p_{ref},T_{ref}\right)\left(p-p_{s}\right)+\;\gamma_{self}\left(p_{ref},\;T_{ref}\right)p_{s}$$

where the calculated pressure p and the measured temperature were used. In Equation (3), the parameter $\gamma_{air}$ is the air-broadened HWHM (cm^−1^), $\gamma_{self}$ is the self-broadened HWHM (cm^−1^), $p_{s}$ is the partial pressure (atm), and *n* is the coefficient of temperature dependence. Equations (1)–(3) were used to model the CM-CLaDS spectrum, and the peak amplitude of the reference signal was used for calibration of the open-path data acquired in a line-locked mode (see [4] for details on the sensor mode of operation). Temperature fluctuations not only affected the calibration channel, but also impacted the precision of the sample channel. In-laboratory testing of the system prior to the field test showed that the main source of drift in long-term measurements was a parasitic fringe between the photodetector window and the telescope body [4]. In the field, when the collection optics were subjected to these large temperatures swings, the effects of the fringe were amplified. From analyzing the noise in the sample channel signals, the estimated precision under these field conditions was determined to be 0.2 ppm (over 40 m and in 1 s). This approximately represents a 5× degradation in system performance; however, despite this increase in fringe noise, the probed path lengths provided enough signal to investigate the feasibility of various sensing configurations in the field.

## 4. Atmospheric Methane Measurements

### 4.1. Diurnal Cycles

**Figure 7:**
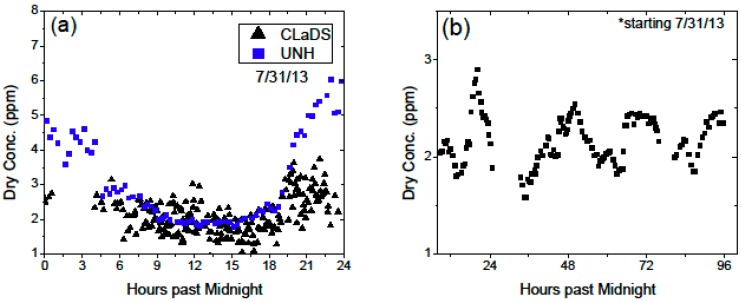
(**a**) Observed diurnal cycle in both the CLaDS system (black) and the University of New Hampshire (UNH) chamber system (blue) on 31 July 2013. Discrepancies in the nighttime peaks are expected due to surface accumulations under the nocturnal boundary layer conditions; (**b**) Diurnal peaks for path #3 of the CLaDS system over 4 consecutive days, starting 31 July 2013.

During the summer months, diurnal cycles of methane near the vegetation floor are expected due to the breakdown of the turbulent mixing layer [19]. During the night hours, the cooler temperatures and reduced wind speeds cause a decrease of this layer height, which prevents transport of emitted methane to the higher portions of the atmosphere. Thus, there is an observed increase in the near-surface methane concentrations during calm nights. Figure 7a shows an example of a 24-h measurement of methane concentration recorded by two independent sensors: the CLaDS instrument and the automated chamber system operated by the University of New Hampshire (UNH) [6,20]. The autochamber setup was sampling air directly from the wetland surface, and air samples were transferred through a tubing system into a commercial spectroscopic instrument (Aerodyne Research, Quantum Cascade Laser Trace Gas Monitor). As shown in Figure 7a both sensors recorded elevated methane levels during the night hours. Different peak concentration values can be explained by the different sampling configurations. The autochamber system was operated directly on the surface, and during the nighttime, the shallow stable boundary layer results in methane accumulation below this layer (seen as up to ~6 ppmv). In the case of the CLaDS instrument, which measured concentration approximately 2 m above the ground, this accumulation is not as clear and much lower diurnal peaks are recorded (~3 ppmv). Figure 7b depicts a four day measurement period of continuous methane monitoring with the CLaDS system, and a diurnal cycle is clearly noticeable in this data.

### 4.2. Multi-Path Measurements

With the sensing configuration described above we have investigated if spatial variations within the fen can be reliably resolved. In low-wind conditions (wind speed < 0.45 m/s, or 1 mph), statistical differences between the path-normalized CH_4_ concentrations measured over different paths were observed indicating differences in emission rates across the fen most likely relating to different plant species underneath the probed optical paths.

**Figure 8:**
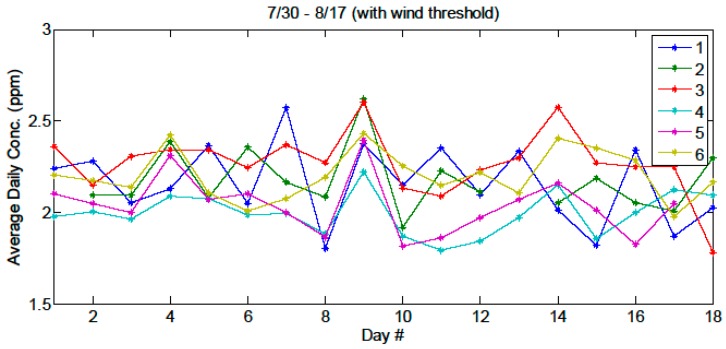
Average daily concentration (path normalized) acquired during low-wind conditions over 17 days of the field campaign. The data clearly illustrates distinct concentration differences between sampled optical paths (indicated with different colors). Rain and thunderstorm events occurred on day 9, and the effect of which can be seen as a clear rise in the emissions from all paths within the fen.

Figure 8 shows data acquired in low-wind conditions for which we assumed that the observed concentrations are related to emissions from plants directly underneath each of the sampled lines of sight. The daily averages for the first 17 days of the measurement campaign shows clear trends for the various optical paths, which can give insight into the emission differences between plant types. For example, paths #3 and #6, which consistently shows a higher daily average, are located in regions dominated by sedges. As seen previously at this site and others, sedge-dominated sites have higher methane emissions than non-sedge sites because sedges are direct conduits of methane from below ground to the atmosphere [7]. In contrast, paths #4 and #5 are located in shrub (non-sedge) abundant areas. This test demonstrates that in low wind-speed conditions the spatial information provided by this single central remote-sensing instrument used in a multi-path configuration makes such studies possible.

At higher wind-speeds the assumption that the measurement represents the emissions from the plants directly below the line of sight is no longer valid. Instead, the wind transports methane from various locations depending of the wind speed, direction, and turbulence. For steady winds the knowledge of the horizontal wind speed and direction can be used in conjunction with the multi-path methane measurement to give insight into large-scale emission trends and potentially locate methane sources. For example, this system can be used to observe events of methane transport and mixing.

**Figure 9:**
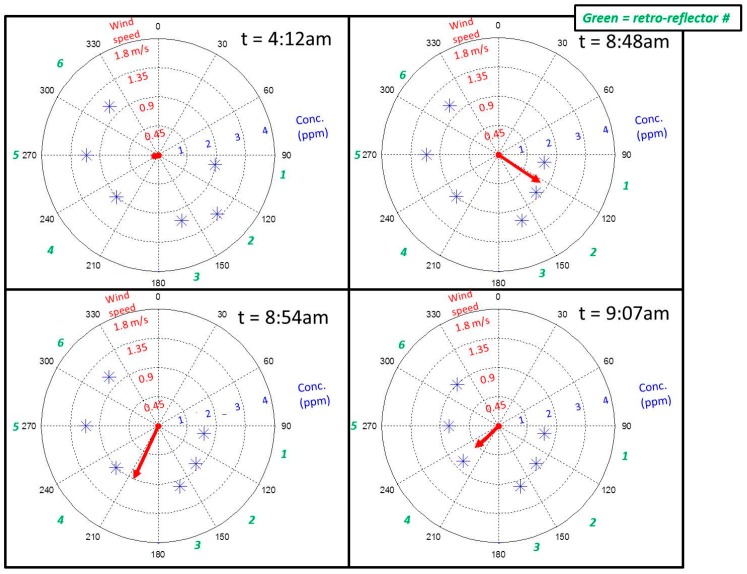
Illustration of observed mixing, and the corresponding decrease in measured CH_4_ concentration, resultant from the onset of winds with speed greater than 0.45 m/s, which on 3 August 2013 occurs just before 09:00, local time. The red vector and blue stars indicate the magnitude and direction for the wind and CH_4_ concentration measurements, respectively, while the green numbers indicate where each sample path is located with respect to the wind direction.

In Figure 9, mixing effects are observed as a result of the breakup of the nocturnal boundary layer as and long-distance transport. Prior to sunrise and the onset of non-zero wind speeds, at 04:12 all paths measured average methane levels greater than 2 ppmv, consistent with the slight nocturnal accumulation mentioned previously. Just before 09:00, the first significant wind of the day generates mixing which allows methane to slowly dilute. Given that the fen is a net emitter of methane the overall mixing with the air masses from outside the fen expectedly results in an observed decrease in the path-normalized concentrations at wind speeds greater than 0.45 m/s. Using this multi-path sensing approach, the temporal and spatial occurrence of this nocturnal boundary layer breakup can be investigated by looking at the influence of wind conditions on various measurement paths. The same approach could be used to study the role of emissions from the fen in the larger-scale atmospheric gas exchange.

## 5. Conclusions

In this work we demonstrated the feasibility of a CM-CLaDS system for open-path sensing of atmospheric methane over multiple sampling paths covering a large area footprint. Operation in the field at SFES provided a challenging environment to test the functionality and robustness of the instrument. A dedicated spectroscopic reference data correction was developed to provide robust calibration of the instrument in the presence of temperature fluctuations within the sensor enclosure. We have also shown that despite operating in a challenging environment with an unstable wetland floor, we have been able to mitigate the opto-mechanical stability issues with a fully automated optical alignment system. In addition, parasitic etalons from the collection optics (*i.e.*, the telescope and detector), were found to be the limiting source of signal drift in our system, and deteriorated the system performance by ~5× with respect to laboratory setting. Despite these challenges, we were able to demonstrate the ability of a remote, multiple-path sensor for investigation of larger-scale methane trends; such as diurnal cycles, the influence of wind dynamics and transport, as well as emissions from different sample paths of various plant species. Further improvements to the sensor will involve a redesign of the free space optics to minimize unwanted interference effects, customization of the sensor electronics to increase functionality and decrease the amount of waste heat within the sensor enclosure.
